# Post-Translational Modifications Drive Success and Failure of Fungal–Host Interactions

**DOI:** 10.3390/jof7020124

**Published:** 2021-02-09

**Authors:** Charmaine Retanal, Brianna Ball, Jennifer Geddes-McAlister

**Affiliations:** Molecular and Cellular Biology Department, University of Guelph, Guelph, ON N1G 2W1, Canada; cretanal@uoguelph.ca (C.R.); ballb@uoguelph.ca (B.B.)

**Keywords:** fungal pathogenesis, host–pathogen interactions, virulence, post-translational modifications, antifungal resistance, anti-virulence

## Abstract

Post-translational modifications (PTMs) change the structure and function of proteins and regulate a diverse array of biological processes. Fungal pathogens rely on PTMs to modulate protein production and activity during infection, manipulate the host response, and ultimately, promote fungal survival. Given the high mortality rates of fungal infections on a global scale, along with the emergence of antifungal-resistant species, identifying new treatment options is critical. In this review, we focus on the role of PTMs (e.g., phosphorylation, acetylation, ubiquitination, glycosylation, and methylation) among the highly prevalent and medically relevant fungal pathogens, *Candida* spp., *Aspergillus* spp., and *Cryptococcus* spp. We explore the role of PTMs in fungal stress response and host adaptation, the use of PTMs to manipulate host cells and the immune system upon fungal invasion, and the importance of PTMs in conferring antifungal resistance. We also provide a critical view on the current knowledgebase, pose questions key to our understanding of the intricate roles of PTMs within fungal pathogens, and provide research opportunities to uncover new therapeutic strategies.

## 1. Introduction

**Figure 1:**
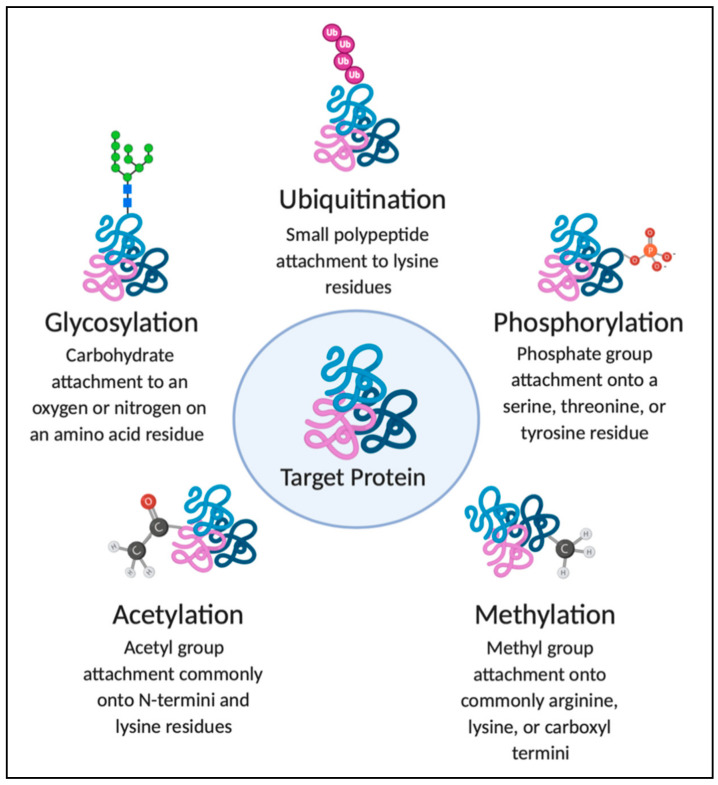
Post-translational modifications highlighted in this review. Fungal proteins can be modified following translation to derive a wide array of unique functions. These diverse modifications may involve covalent or noncovalent additions of polypeptides or proteins, complex molecules, chemical groups, as well as proteolysis. The primary PTMs discussed in this review include phosphorylation, acetylation, deacetylation, ubiquitination, and glycosylation.

Post-translational modification (PTM) of proteins is essential for many biological processes, including environmental adaptation, cell growth and division, development, and survival. PTMs change the properties of proteins, which provide them with the specific functional and/or structural propensities necessary to drive foundational processes in a cell, thereby increasing the diversity of the proteome. PTMs include covalent or noncovalent addition of a functional group to a protein, cleavage of proteins by peptidases or proteases, or complete degradation of a protein [1]. Such modifications encompass the events of phosphorylation (addition of a phosphoryl group), acetylation (introduction of an acetyl functional group), ubiquitination (attachment of a ubiquitin protein), glycosylation (addition of glycosyl donor), methylation (addition of methyl group), and others. These modifications, including the processes and outcomes, are explored in Figure 1. Microbial pathogens use PTM mechanisms to regulate protein production and activity during infection and manipulate host proteins for disease progression [2,3,4]. Specifically, fungal pathogens exploit PTMs to enhance pathogenicity and promote fungal survival [1,5].

Fungal pathogens are critical threats to global health, with more than 300 million people afflicted with serious fungal diseases annually [6]. In recent years, the frequency of invasive fungal infections has increased by over 200%, with patient mortality rates ranging from 30 to 90% [7]. Fungal infections are particularly prevalent among immunocompromised individuals, including those suffering from HIV/AIDS, cancer patients receiving immunotherapy, organ transplant recipients administered immunosuppressive drugs, as well as the elderly population [8]. Treatment of fungal infections is a serious challenge with a limited selection of low host toxicity, clinically effective antifungal drugs, and the emergence of resistant strains [9,10,11,12]. Among medically relevant fungal pathogens, *Candida* spp., *Aspergillus* spp., and *Cryptococcus* spp. are the most prevalent opportunistic fungi that cause infections worldwide in immunocompromised or immunosuppressed patients, causing mortality [13]. These fungal pathogens result in the mortality of over one million people annually and cost billions in treatment, representing a substantial economic burden [14].

Among *Candida* spp., *Candida albicans* is a polymorphic fungus capable of morphological switching, which contributes to its virulence, and is part of the normal human microbiota [15]. *C. albicans* is responsible for most superficial mucosal infections and causes different types of candidiasis (e.g., thrush, yeast infections) through overgrowth in the human microbiota, leading to inflammation of the host [16]. *Cryptococcus* spp., primarily, *Cryptococcus neoformans* and *Cryptococcus gattii*, are yeast-like fungi found ubiquitously within the environment, capable of causing disease in immunocompromised and immunocompetent individuals, respectively [8,17]. The fungus relies on the production of numerous virulence factors, including a polysaccharide capsule, melanin, extracellular proteases, and thermotolerance, to cause fatal cases of meningoencephalitis [18,19]. For *Aspergillus* spp., *Aspergillus fumigatus* is a filamentous mould found primarily in soil and decaying vegetation, with infection occurring through inhalation of conidia [20]. Opportunistic infections may lead to severe manifestations of invasive pulmonary aspergillosis [21]. The occurrence of PTMs within these fungal pathogens influence virulence factor production and the outcome of infections.

To improve our ability to combat fungal infections, it is critical to define how pathogens use PTMs to maintain and modify cellular processes, adapt to the host cell environment, and mediate the host response to infection. In this review, we explore the impact of PTMs, including phosphorylation, acetylation, glycosylation, ubiquitination, and methylation used by fungal pathogens. Specifically, we address the role of PTMs in stress response and host adaptation, discuss how fungi use PTMs to manipulate host cells and evade the immune response, and present how PTMs contribute to antifungal resistance. We focus on scientific advances over the past decade and propose future investigations to enhance the expanding body of literature focusing on PTMs of microbial pathogens to provide scientists with opportunities to uncover novel therapeutic strategies.

## 2. PTMs in Stress Response and Host Adaptation

**Figure 2:**
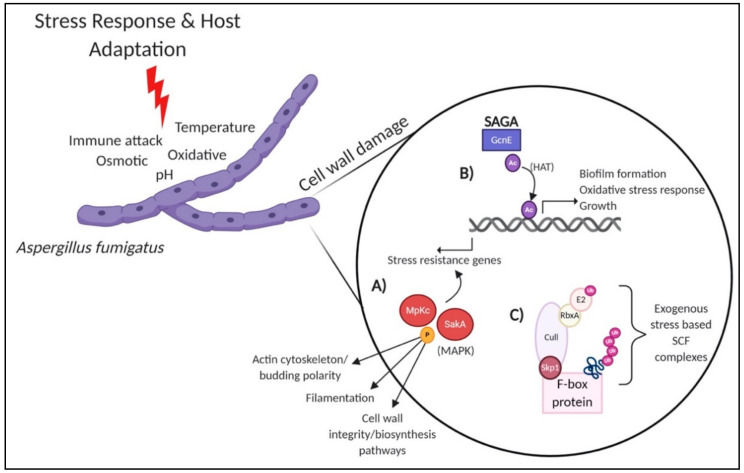
PTMs involved in cell wall stress responses within *Aspergillus fumigatus.* (**A**) SakA and MpkC, main regulators of the HOG pathway and MAPKs, are signaling proteins that control novel downstream phosphorylation events of protein kinases and transcription factors involved in tolerance to the cell wall damage response and cell wall integrity pathway [29]. (**B**) The acetyltransferase, GcnE, contributes to gene regulation for oxidative stress responses through the action of histone modification [30]. (**C**) The SCF complex regulates protein degradation in fungi. Novel F-box and SCF complex conformations identified in response to various exogenous stresses, including cell wall and oxidative stress are depicted [31]. MAPK: mitogen-activated protein kinase, HAT: histone acetyltransferase, SAGA: Spt-Ada-Gcn5 acetyltransferase, and SCF: Skp1-Cul1-F-box complex.

The fungal cell wall is the first point of contact between the pathogen and host, and the ability of fungal cells to survive within the host environment depends on the function, organization, and composition of the cell wall (Figure 2). The cell wall provides protection through activation of multiple pathways in response to stress stimuli, including osmotic stress, pH change, temperature change, or drug-induced stress [22,23]. Responses to such stresses include biofilm formation, capsule enlargement, filamentation, and melanin production, all of which, are key fungal virulence factors. Integrity of the cell wall is maintained through numerous kinase-dependent pathways, for example, the cell wall integrity (CWI) pathway, which controls cell wall biosynthesis and repair, the high osmotic glycerol (HOG) pathway, which regulates cellular response to osmotic stress, and the mitogen-activated protein kinase (MAPK) signaling pathway, which is crucial for adaptation to the environment [22,24,25,26,27]. Alternatively, the calcineurin (protein phosphatase) signaling pathway regulated by intracellular Ca^2+^, also plays a role in stress response [28].

Activation of cell wall-associated pathways via phosphorylation drives fungal tolerance to the host environment. For example, in *A. fumigatus*, global phosphoproteome profiling revealed the fungal response to cell wall stress generated with Congo red (cell wall-damaging agent), and determined 485 proteins potentially involved in the cell wall damage response [29]. The study focused on the role of two MAPKs: SakA and MpkC, main regulators of the HOG pathway with crucial roles in antifungal agent (e.g., caspofungin) tolerance (Figure 2A). Proteins phosphorylated during cell wall stress were involved in signal transduction, stress response, protein kinases, actin cytoskeleton and budding cell polarity, and filamentation. The study highlighted the involvement of the HOG pathway in cell wall stress response and identified several novel proteins important for cell wall preservation, suggesting new components of fungal signaling events that may serve as novel targets for antifungals. Future work proposes the characterization of osmotic and cell wall stress kinases and transcriptional factors as an opportunity for interference with crucial fungal signaling events.

Fungal stress response is also regulated through the action of acetyltransferases, which drive the PTM of acetylation. A relationship was recently discovered between the histone acetyltransferase (HAT), GcnE, and important *A. fumigatus* development and regulatory processes, including growth, biofilm formation, and tolerance to stress (Figure 2B) [30]. The acetyltransferase, Gcn5 (GcnE in *A. fumigatus*), is part of the evolutionarily conserved Gcn5-related N-acetyltransferase family (GNATs) and the large transcriptional multiprotein complex Spt-Ada-Gcn5 acetyltransferase (SAGA) [32]. A previous study showed that deletion of *gcnE* in the phytopathogen *Aspergillus flavus* resulted in increased susceptibility to cell wall stress-inducing agents (e.g., calcofluor white and Congo Red) [33]. In the present study, loss of *gcnE* led to significant sensitivity to Congo Red and sodium dodecyl sulfate (SDS) compared to the wild-type *A. fumigatus* [30]. Additionally, the *gcnE*Δ mutant also showed susceptibility to oxidative stress but had a higher tolerance against osmotic stress than the wild type. Overall, GcnE is required for oxidative stress response, but not osmotic stress response, suggesting targeted opportunities to regulate fungal defenses through acetylation events.

In *C. albicans*, Gcn5 affects stress response, similar to GcnE in *A. fumigatus*, and also influences fungal morphology and pathogenicity [34]. Recently, a connection between phosphorylation and acetylation was observed through the impact of Gcn5 on the MAPK pathway [32]. The authors showed that homozygous *gcn5*Δ/Δ mutant cells demonstrated a decrease in phosphorylated Mkc1 activity, but an increase in the activity of phosphorylated Cek1 and Hog1 compared to the nonphosphorylated Mkc1, Cek1 and Hog1 controls. These data indicate that the absence of Gcn5 differentially regulates MAPK signaling and cell wall components, as well as glucan synthases (e.g., *FKS*), cell adhesion, and hypersensitivity to host-derived oxidative stress, which play critical roles in virulence and antifungal susceptibility. In *C. neoformans*, Gcn5 is critical for fungal adaptation to the host environment by regulating high-temperature growth, capsule attachment to the fungal cell surface, and providing protection against oxidative stress [35]. When exposed to high temperatures (i.e., 37 °C), the *gnc5*Δ strain showed a delay in growth relative to the wild type; however, no growth defects were observed at 30 °C. Furthermore, a reduction in response to oxidative stress was observed for the mutant strain but exposure to the permeabilizing agent, SDS, did not affect fungal stability. Taken together, these studies highlight that similarly to *A. fumigatus* and *C. albicans*, *C. neoformans* uses the HOG1/MAPK pathways, along with the Calcineurin pathway, to regulate genes during osmotic and heat stress. 

Another PTM involved in stress response and environmental adaptation includes ubiquitination. In *C. albicans*, molecular, cellular, and proteomic profiling approaches were combined for an integrative analysis of *UBI4*, a ubiquitin gene encoding for a polyubiquitin involved in hyphae and pseudohyphae growth, temperature sensitivity, and stress response [36,37]. Here, the construction of independent null and conditional mutants for *ubi4* produced morphological and cell cycle defects, sensitivity to thermal, oxidative, and cell wall stress, and proteomic identification of 19 ubiquitination targets with roles in growth, stress response, and metabolic adaptation related to *UBI4* function [37]. This study uncovered a connection between polyubiquitin and fungal virulence and suggests a potential novel target for antifungal development. Additional studies have explored the impact of the ubiquitin proteasome on protein turnover and degradation, as well as connections to major regulatory systems. For instance, in *C. neoformans*, a connection between the cAMP/protein kinase A (PKA) signaling cascade (associated with virulence factor elaboration) and the ubiquitin proteasome uncovered a novel drug-repurposing strategy to inhibit production of the polysaccharide capsule and reduce fungal virulence [38]. Furthermore, investigation into the association of ubiquitination and degradation of proteins bound by F-box proteins in *A. fumigatus* and *A. flavus* defined the composition of F-box and scaffold protein complex (Skp1-Cul1-F-box (SCF) complex) [31]. This study identified novel interactions with F-box proteins during the formation of the SCF complex, demonstrated varied responses of different SCF complex conformations to exogenous stresses, including cell wall and oxidative stress, and defined a connection between F-box interaction partners and antifungal resistance (Figure 2C). Additional studies explored F-box proteins of the ubiquitin–proteasome system to describe a role for Fbp1 in regulating fungal–macrophage interaction and fungal virulence, as well as shaping immunogenicity, demonstrating the multidimensional layers of ubiquitination control within fungal pathogens [39,40]. The role of F-box proteins and their potential as novel antifungal targets will be explored further in the sections below.

Methylation (the addition of a methyl group to a protein) is connected with regulation of toxic secondary metabolites in fungal pathogens, including *A. fumigatus*. In filamentous fungi, toxic secondary metabolite production is mechanistically associated with chromatic remodeling and the action of CclA, a member of the histone 3 lysine 4 (H3K4) methylating COMPASS complex, which suppresses secondary metabolite production [41,42]. Deletion of *cclA* resulted in tri- and di-methylation deficiency of H3K4 and yielded a slow growing fungal strain, rich in production of several secondary metabolites (e.g., gliotoxin), with comparable virulence to the wild type in a murine model of invasive aspergillosis [43,44]. The data support the role of methylation in regulating secondary metabolites, which influence fungal pathogenicity. Taken together, PTMs play critical roles in preparing fungal cells for high-stress environments, such as those encountered during infection of the host through regulation of signaling cascades, protein degradation, and protein activation, and more commonly, studies are exploring the interconnectivity of these mechanisms.

## 3. PTMs for Host Cell Manipulation and Immune System Evasion

**Figure 3:**
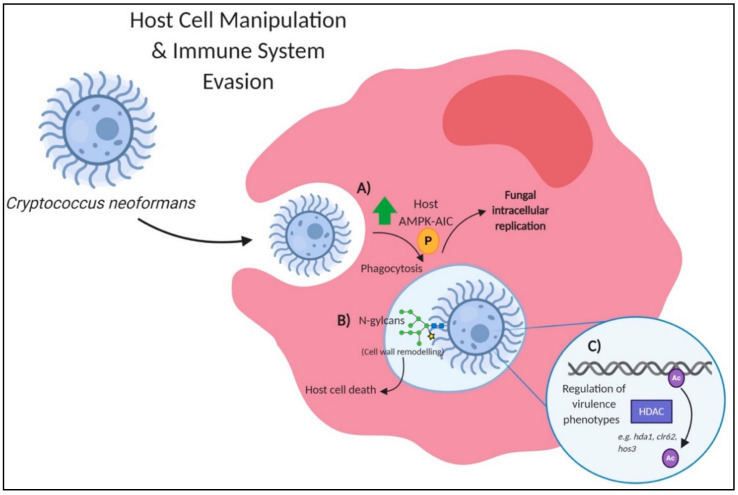
PTMs involved in host–fungi interactions within *Cryptococcus neoformans.* (**A**) Global reprogramming of host kinase cascades occurs upon fungal invasion. Phagocytosis of *C. neoformans* activates the host AMPK-AIC signaling network to regulate intracellular trafficking and replication of the pathogen [45]. (**B**) Internalized *C. neoformans* cell wall modeling exposes core *N*-glycans, which triggers host cell death, and subsequent fungal escape [48]. (**C**) HDAC activity results in repression of gene transcription networks via the removal of acetyl groups; this results in regulation of *C. neoformans* main virulence phenotypes, including melanin, capsule production, protease activity and cell wall integrity [49]. AIC: autophagy initiating complex; HDAC: histone deacetylase.

To prevail during infection, a fungus needs to adapt to its environment and also evade the host immune response (Figure 3). The ability to manipulate host cells and prevent their inherent response is essential for the progression of fungal disease. Recently, differential phosphorylation analysis of host cells infected with *C. neoformans* revealed reprogramming of the host autophagocytic response [45] (Figure 3A). Similar findings of induced pro-inflammatory and anti-apoptotic signals in macrophages were observed through phosphoproteome profile of *C. albicans* [46]. During fungal invasion of the host, the immune system works to rid the host of foreign invaders through the action of autophagy to remove damaged cells. The autophagy initiating complex (AIC) is important for the early stages of autophagy induction. The AMP-activated protein kinase (AMPK) and mammalian target of rapamycin complex (mTORC1) complexes regulate autophagy by sending opposing signals to the AIC, where AMPK positively regulates autophagy induction through inhibition of mTORC1 [47]. When infected with *C. neoformans*, the host AMPK-AIC signaling network is differentially phosphorylated [45]. The study further showed that phagocytosis of *C. neoformans* by macrophages activates the AIC and the phosphorylation of ULK1, LKB1 and AMPKα. The AIC controls the internalization of *C. neoformans* into host cells by recruiting AIC components to form phagosomes and promotes *Cryptococcus*-containing vacuole biogenesis, enabling intracellular replication of the pathogen. Therefore, once phagocytosis of *C. neoformans* occurs, host phosphorylation of LKB1 kinase activates AMPK, which phosphorylates the ULK1 kinase in AIC, allowing susceptibility to *C. neoformans* infection. This study highlights the evolved phenomenon of fungal pathogens’ propensity to advantageously manipulate host signaling networks to maintain intracellular survival and subsequent host dissemination.

Other approaches to evading immune cells include induction of host cell death, such as macrophage pyroptosis, an inflammatory cell death program [50]. For example, phagocytosis of *C. albicans* by macrophages triggers a morphological switch from yeast to filamentous form, a key virulence factor that influences the activation of pyroptosis [51]. Notably, pyroptosis is not activated by filamentation, but rather the process of fungal cell wall modeling [52,53]. A recent study using high-throughput screening of a tetracycline-repressible conditional expression strain in *C. albicans* defined the requirement for Hog1 in cell wall modeling during macrophage internalization, as Hog1 signaling is needed for regulation of cell wall factors and mannose exposure [54]. Two-component signaling kinases were identified to be required for macrophage pyroptosis, including SLN1 (controls activity of the HOG1 pathway), CHK1 (histidine kinase that regulates cell wall mannan and glucan biosynthesis), and NIK1 (histidine kinase that regulates cell wall mannan biosynthesis). PTMs modulate the pathogen’s morphological change, adding pressure on the phagosome, causing it to rupture, resulting in macrophage pyroptosis, which allows the pathogen to escape, and ultimately evade the host immune response. Overall, this study demonstrates the role of cell wall modeling to activate the host inflammasome.

*C. neoformans* also causes macrophage pyroptosis, but in a slightly different manner to *C. albicans*, as described above. *C. neoformans* causes physical stress to the phagosomes through enlargement of its polysaccharide capsule, a key virulence factor mediated through phosphorylation via the cAMP/PKA pathway, CWI, and HOG1 pathways [55,56,57]. When exposed to the phagosome’s low pH, *C. neoformans* undergoes cell wall modeling to increase capsule size, provide protection to the pathogen, and cause stress on the phagosomal lipid membrane [58]. If the phagosomal membrane is ruptured, activation of the host inflammasome occurs, causing macrophage pyroptosis. Investigation of histone deacetylation, a regulated PTM opposing the action of histone acetylation, demonstrated reduced control of cellular processes associated with virulence, including thermotolerance, capsule formation, melanin synthesis, protease activity, and cell wall integrity [49,59]. Recently, deleting histone deacetylases (HDACs) involved in capsule elaboration (e.g., *hda1*Δ, *clr62*Δ and *hos3*Δ) showed a reduction in capsule enlargement when exposed to capsule-inducing conditions [49] (Figure 3C). Notably, *hos1*Δ showed a larger capsule than wild-type *C. neoformans*, indicating opposing functions of HDACs on capsule enlargement. The study also showed that deletion of HDACs (e.g., *hda1*Δ, *hos1*Δ, *clr62*Δ, *hos2*Δ, and *rpd3*Δ) reduced fungal survival within macrophages. Overall, this work highlights the importance of PTM regulation to control fungal virulence factor production and pathogenicity, determining fungal survivability during an encounter with the host.

Glycosylation is another important PTM that is crucial for functional regulation of the fungal proteome, including protein activity, folding, stability, transport, and immunogenicity [60]. For fungal pathogens, glycans (*N*- and *O*-linked) assemble on cell surface glycoproteins to regulate pathogen adhesion and interaction with host cells during infection [61]. The critical role of glycosylation in cell wall integrity, morphogenesis, virulence, and immune recognition has been reported in *Candida* spp., *A. fumigatus*, and *Histoplasma capsulatum* [62,63,64,65]. In a recent study, the importance of core *N*-glycan structure in relation to the virulence of *C. neoformans* was explored [48] (Figure 3B). Here, the authors modified the assembly of the core *N*-glycan by targeted gene deletions of specific glycosyltransferases, a part of the asparagine-linked glycosylation (ALG) pathway. The production of truncated neutral *N*-glycans by deleting *ALG3* resulted in a reduced ability of the fungal cells to drive macrophage cell death and attenuated virulence *in vivo*, supporting the role for *N*-glycans in promoting host cell escape and fungal dissemination. Another study also explored the importance of glycosylation in pathogenesis of *C. albicans* by investigating the PTMs role in phagocytosis, hyphal formation, and escape from macrophages [66]. The study determined that cell wall glycosylation is important for recognition and ingestion of the fungal pathogen by macrophages. Moreover, a diminished ability of glycosylation mutants to kill macrophages supports the role of *O*- and *N*-linked glycans in proper cell wall composition, critical for fungal survival within the host. A similar role for glycosylation and cell wall integrity was defined for modulation of epithelial immunity and apoptosis induction in *C. albicans* [67]. These studies exploit the importance of glycosylation on fungal pathogenicity with primary immune cells and emphasize the role of proper cell wall composition for fungal dissemination and survival. Overall, this section highlights a diverse array of PTMs that modulate critical mechanisms used by fungal pathogens to manipulate the host and evade the immune response in promotion of their own survival.

## 4. PTMs Influence Antifungal Resistance

**Figure 4:**
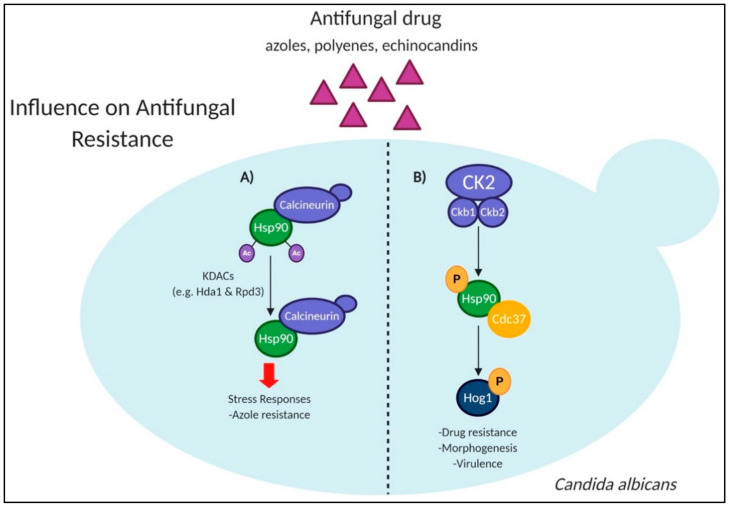
PTMs involved in antifungal resistance within *Candida albicans.* (**A**) Acetylation maintenance governed by KDACs controls the function of Hsp90 with client proteins, such as the drug-resistance regulator calcineurin, to mediate azole resistance [73,74]. (**B**) The regulatory subunits of protein kinase CK2 regulate the function of Hsp90 along with the co-chaperone Cdc37 by phosphorylation, which impacts the stability of downstream interactors, including the kinase Hog1 [75]. KDAC: lysine deacetylase; Ckb1/2: regulatory subunits of protein kinase CK2.

Current antifungal treatments demonstrate limited efficacy against the evolution and emergence of antifungal resistance [9,10]. In addition, the development of new antifungal agents is challenging due to analogous eukaryotic targets between host and pathogen, as well as issues with prolonged treatment regimens and host toxicity. Importantly, alteration of proteins by PTMs can be exploited by fungal pathogens to confer antifungal resistance (Figure 4). For example, heat shock protein 90 (Hsp90), a molecular chaperone mediated by PTMs, regulates diverse cellular processes, including folding and maintaining substrate proteins, and it is critical for developing antifungal resistance [68,69,70,71,72]. Given its role in regulating fungal virulence factors, Hsp90 presents an attractive target for inhibition as a treatment for controlling fungal infection; however, the similarity between fungal and host targets confers host toxicity, limiting its efficacy. Therefore, determining how Hsp90 is regulated by PTMs, and the network of proteins mediating resistance, can provide insight into potential new targets for antifungal drugs.

In *C. albicans*, Hsp90 is a key controller of virulence, and acetylation of Hsp90 through the action of lysine deacetylases (KDACs) is a vital regulator of its function, suggesting a role for KDACs in antifungal resistance in *C. albicans* [73] (Figure 4A). Investigation of redundancy among KDACs was defined for Hos2, Hda1, Rpd3, and Rpd31 through their mediation of azole resistance and morphogenesis [74]. Specifically, Hos2 positively regulated Hsp90 function, and removal of the KDAC caused enhanced filamentation in the absence of a proper induction cue. The *hda1*Δ, *hos2*Δ, *rpd3*Δ, and *rpd31*Δ strains individually abolished azole resistance, and the study determined that proper function of Hsp90 is driven through critical acetylation sites (lysine 30, 271). A complementary study featuring an acetylome comparative analysis revealed an interspecies contrast between nonpathogenic *S. cerevisiae* to highly virulent *C. neoformans*, *A. fumigatus*, and *C. albicans*. Essential roles for acetylation in these fungal pathogens were identified and determined that protein acetylation levels correlate to fungal pathogenicity [76]. Here, the authors identified 159 genes co-regulated by KDACs, Dac2 and Dac4, key regulators of fungal virulence, and revealed highly dynamic features of acetylation within fungal species. This information can help develop antifungals by determining the evolutionarily conserved protein lysine acetylation sites and, subsequently, targeting these sites for broad antifungal treatment against *C. albicans*, *C. neoformans*, and *A. fumigatus*. 

Multiple PTMs can regulate proteins at different sites to influence activity and function. For example, in *C. albicans*, Hsp90 is phosphorylated by regulatory subunits of kinase CK2, impacting the stability and function of Hsp90 targets, including Hog1, which is necessary for stress response [75] (Figure 4B). Another example encompasses the role of CWI components (e.g., PkcA, MpkA, and RlmA), which interact with Hsp90 during heat shock and cell wall stress adaptation [77]. PkcA is a critical kinase in the CWI pathway and necessary for fungal survival during exposure to antifungal drugs [78]. In the current study, mutations in the C1B domain of PkcA prevented interaction with Hsp90, abolishing the CWI pathway response and supporting the potential of PkcA as a novel antifungal target; however, similarity with the host PKCβII protein demonstrates cross-reactivity and blocking [77]. These findings determined that CWI proteins are Hsp90 substrates, and Hsp90 stabilizes the CWI proteins, regulating response to heat stress. Despite such findings, the comprehensive network of Hsp90 has yet to be elucidated, and the mechanisms underscoring interactions between Hsp90 and its substrates to regulate antifungal resistance requires further exploration.

Stress kinases involved in phosphorylation can also confer resistance to current antifungals. For example, in *C. albicans*, it was recently reported that inhibition of the stress kinase, Yck2, conferred resistance of the fungal pathogen to the echinocandin, caspofungin [79]. This study screened a library of protein kinase inhibitors for the ability to reverse resistance and identified multiple 2,3,-aryl-pyrazolopyridine scaffold compounds capable of restoring caspofungin sensitivity. Chemical genomic, biochemical, and structural studies identified the Yck2 as the compound target, *in vitro* studies demonstrated biosafety in the presence of human cells, and *in vivo* murine model assessment confirmed the role of the kinase in fungal survival. Taken together, this study highlights a critical kinase associated with antifungal resistance and compound screening extrapolates this information to identify new therapeutic tools to combat fungal infection. 

## 5. Perspective and Future Outlook

Despite the formidable progress made to date defining the roles of PTMs in fungal regulation, further research to comprehensively understand the complexity of biological processes modulated by the PTMs described herein, and the cross-talk amongst such regulation, is still needed. For instance, although a connection between KDACs and governance of azole resistance in *C. albicans* has been defined, an interaction between multiple KDACs and the possibility of site hierarchy or redundancy when interacting with Hsp90, is not understood [74]. Future research into the specific interactions and potential compensatory roles may assist in the design of KDAC inhibitors for combinatorial treatments [80]. Further, given the conservation of Hsp90 and its roles among biological systems, defining fungi-specific targets may prove effective against *C. albicans* and other fungal pathogens. The challenge of identifying fungi-specific targets (distinct from common human targets) is a substantial hurdle faced in antifungal drug design and development. Recently, investigation of metabolic routes (e.g., trehalose and amino acid metabolism) and metabolic networks used by fungi during infection, along with molecules inhibiting growth, enzymatic inhibitors, and anti-virulence strategies, as well as computer modeling, show promise in expanding our repertoire of antifungals [81,82,83,84,85,86]. Other studies focus on the precise impacts of PTM localization, such as acetylation sites at lysine 30 and 271 on Hsp90 in *C. albicans*, which were determined critical for mediating Hsp90-dependent azole resistance; however, these residues were analyzed based on reports in *S. cerevisiae* and *A. fumigatus*. Further investigation into potential fungi-specific acetylation sites with Hsp90 may uncover tailormade opportunities for inhibition of specific fungal pathogens and potentially uncover new sites for inhibition.

Another notable area of future discovery is profiling the impact of microbial infections from dual perspectives (i.e., how does the host respond to infection and how does the pathogen evade the host defenses) [3,87,88]. Characterization of the host phosphoproteome-regulated response to cryptococcal infection defined reprogramming of host kinase signaling in murine models based on opsonization of the fungal pathogen [45]. A limitation is exploring the natural interaction between host and pathogen lacking experimental opsonization to promote fungal uptake, which would provide a clearer picture of the infection process. Moreover, the study does not report phosphoproteome changes of the pathogen during infection, a relatively new area of study currently being explored by our research group and others [87]. Furthermore, during the course of infection, the host responds to initial invasion, mounts a defense, and maintains a heightened level of immunity in the event of chronic infection or a future encounter with the pathogen; therefore, profiling a time course of infection may provide a comprehensive picture of host response and reveal time- or location-dependent opportunities for new therapeutic tools [4].

With the abundance of research conducted on the comparative analysis of kinases and (de)acetylases in fungal pathogen virulence, more information on such enzyme characterization may identify new targets for antifungal treatments. For example, a high-quality library consisting of signature-tagged gene deletion strains of 129 putative kinases in *C. neoformans* revealed 63 pathogenicity-related kinases as potential targets for inhibition through anti-virulence strategies [89]. However, despite such extensive characterization and coverage of fungal kinases, over 50 candidates selected based on orthologous genes essential for growth in *Saccharomyces* spp. were not explored due to a lack of viable transformants or incorrect genotype, or persistence of the wild-type allele, suggesting that alternative strategies, such as interference CRISPR, may have application in *C. neoformans* [90,91]. Moreover, the application of anti-virulence therapeutics (i.e., antimicrobial agents that target virulence factors produced by the pathogen rather than the pathogen itself) supports clearance of the pathogen from the host while limiting the evolution of resistance [12,92].

In a broader context, identifying kinases responsible for phosphorylating specific proteins is difficult and a main limitation in understanding mechanisms of phosphorylation. Recent technological advances in mass spectrometry-based phosphoproteomics have improved our ability to detect and quantify the global impact of phosphosites, providing new insight into phosphorylation networks [93]. Moreover, bioinformatics tools are critical for predicting kinases associated with specific phosphorylation events, as well as individual phosphosites; however, experimental validation is required for confirmation of *bona fide* biological activity [94]. In addition, bridging a connection between a single kinase and multiple phosphosites is challenging given the often unrestrained and transient nature of the interactions. Despite these limitations, using the developed technological and bioinformatics strategies for phosphoproteomics aids in our understanding of other PTMs, including acetylation, ubiquitination, glycosylation, and methylation, while also informing on the complementary role of phosphatases in fungal pathogens [90]. Taken together, we are propelling our understanding of dynamic and complex biological systems through comprehensive profiling of PTMs to define how the pathogen adapts to the hostile environment of the host and uncover how the host defends itself from the invading pathogen. In the same light, we use information about the mechanisms employed by PTMs to regulate proteins and diverse biological functions as potential new targets for antifungal strategies; however, we still have a long way to go to bring together all of the information about a single pathogen, design new drugs with fungi-only specificity, and extrapolate these findings to multiple medically relevant, globally important fungal threats.

## 6. Conclusions

Considering the amount of information coming forth regarding PTMs in fungal pathogens, there is still a large gap in our understanding of these biological and biochemical phenomena. First, a disconnect between how the various PTMs interact in the same, or opposing pathways, exists. How does such complementary regulation influence protein function and network activity? Secondly, a comprehensive analysis of all PTM sites within a single protein and/or regulatory network remains to be defined. How are multiple sites regulated? During which circumstances are they modified? And how do different PTMs within a single protein impact function? Lastly, understanding how the host system reacts to the pathogen should also be analyzed. What proteins are differentially modified due to fungal invasion? Does timing of infection affect our observations of PTMs driven by the host response? How do these events lead to a successful pathogenic invasion and a failure of the host to provide protection? Given all of these considerations, the advancement of new technologies (e.g., mass spectrometry-based proteomics) and bioinformatics platforms lay the foundation for detection and characterization of PTMs to enhance our understanding of the relationships between a host and fungal pathogen during infection and these continue to increase the complexity of the biological systems and questions that we are asking.
